# Supplementary material for: A Systematic Comparative Study on the Physicochemical Properties, Volatile Compounds, and Biological Activity of Typical Fermented Soy Foods
Source: Foods. 2024 Jan 27;13(3):415. doi: 10.3390/foods13030415 (PMC10855112; doi:10.3390/foods13030415)
Supplement: Supplementary file 1 [file foods-13-00415-s001.zip › foods-2839647-supplementary.pdf]

Table S1. Volatile compounds in different fermented soy foods detected by HS-SPME-GC×GC-MS.

CAS.N	Name	Relative content (%)	CAS.N	Name	Relative content (%)
**Furu**			**Douchi**		
123-66-0	Ethyl caproate	12.023±11.993	2258-42-6	Formyl acetate	13.701±9.366
106-32-1	Ethyl caprylate	9.416±7.052	5617-32-3	Heptaethylene glycol	5.589±4.058
64-17-5	Ethanol	7.878±7.792	5117-19-1	Octaethylene glycol	4.972±3.048
629-59-4	Tetradecane	6.302±6.322	3055-98-9	Octaethylene glycol lauryl ether	3.334±3.080
5617-32-3	Heptaethylene glycol	4.397±1.384	56599-95-2	2-Bromooctadecanal	2.649±3.698
104-46-1	cis-Anethol	3.143±3.650	513-85-9	2,3-Butanediol	2.516±1.793
5117-19-1	Octaethylene glycol	2.321±0.481	100-52-7	Benzaldehyde	2.494±1.214
541-05-9	Hexamethylcyclotrisiloxane	2.090±0.909	541-05-9	Hexamethylcyclotrisiloxane	2.386±1.719
628-97-7	Ethyl palmitate	1.304±0.180	105-43-1	3-Methylvaleric acid	2.342±1.551
3391-86-4	1-Octen-3-ol	1.238±0.105	1066-42-8	Dimethylsilanediol	2.202±0.393
3055-98-9	Octaethylene glycol lauryl ether	1.233±0.270	108-50-9	2,6-Dimethylpyrazine	1.888±1.255
1066-42-8	Dimethylsilanediol	1.168±0.185	5469-16-9	Dihydro-4-hydroxy-2(3H)-furanone	1.421±1.712
71-41-0	1-Pentanol	0.685±0.553	3268-49-3	3-(Methylthio)propionaldehyde	1.310±1.438
821-55-6	2-Nonanone	0.626±0.333	110-93-0	6-Methyl-5-hepten-2-one	1.294±1.288
140-67-0	4-Allylanisole	0.575±0.669	122-78-1	Phenylacetaldehyde	1.261±0.682
100-52-7	Benzaldehyde	0.554±0.271	64-19-7	Acetic acid	1.197±0.113
108-95-2	Phenol	0.530±0.472	71-41-0	1-Pentanol	1.066±0.749
110-43-0	2-Heptanone	0.516±0.104	2615-15-8	Hexaethyleneglycol	0.746±0.487
122-78-1	Phenylacetaldehyde	0.516±0.298	13887-98-4	3,6,9-Trioxaundecanedioic acid	0.661±0.641
5353-25-3	2-[(Z)-9-Octadecenyloxy] ethanol	0.514±0.389	646-07-1	4-Methylpentanoic acid	0.552±0.195
661-19-8	1-Docosanol	0.487±0.571	624-92-0	Dimethyl disulfide	0.463±0.271
66-25-1	Hexanal	0.434±0.311	3391-86-4	1-Octen-3-ol	0.439±0.140
3777-69-3	2-Pentylfuran	0.364±0.108	21834-92-4	2-Phenyl-5-methyl-2-hexenal	0.422±0.139
105-54-4	Ethyl butanoate	0.359±0.220	4356-52-9	Glucobrassicin	0.421±0.273
111-62-6	Ethyl oleate	0.355±0.072	56-81-5	Glycerol	0.396±0.259
2615-15-8	Hexaethylene glycol	0.350±0.175	556-67-2	Octamethylcyclotetrasiloxane	0.383±0.137
556-67-2	Octamethylcyclotetrasiloxane	0.324±0.296	644-30-4	Alpha-Curcumene	0.315±0.366
513-85-9	2,3-Butanediol	0.291±0.097	598-75-4	3-Methyl-2-butanol	0.244±0.185
104-76-7	2-Ethylhexanol	0.287±0.246	26643-91-4	4-Methyl-2-phenyl-2-pentenal	0.213±0.134
106-30-9	Ethyl heptanoate	0.268±0.054	3658-80-8	Dimethyl trisulfide	0.209±0.107
110-44-1	Sorbic acid	0.200±0.177	66-25-1	Hexanal	0.199±0.034
447412-24-0	2-Amino-4,4,6,6-tetramethylthieno [2,3-c] furan-3-carbonitrile	0.188±0.070	108-95-2	Phenol	0.194±0.116
55282-12-7	3-Ethyl-5-(2-ethylbutyl) octadecane	0.180±0.031	3055-97-8	Dodecylheptaglycol	0.192±0.148
55124-79-3	9-Hexylheptadecane	0.177±0.096	600-14-6	2,3-Pentanedione	0.192±0.081
108-64-5	Ethyl isovalerate	0.166±0.096	3857-25-8	5-Methyl-2-furanmethanol	0.192±0.028
97-62-1	Ethyl isobutyrate	0.164±0.085	56666-38-7	2-(12-Pentadecynyloxy) tetrahydro-2H-pyran	0.158±0.041

625-48-9	2-Nitroethanol	0.151±0.163	55282-12-7	3-Ethyl-5-(2-ethylbutyl) octadecane	0.150±0.152
110-38-3	Ethyl caprate	0.147±0.047	17151-09-6	1,2-Bis(trimethylsilyl)benzene	0.110±0.044
589-98-0	3-Octanol	0.139±0.017	628-97-7	Ethyl palmitate	0.110±0.090
98-00-0	Furfuryl alcohol	0.120±0.114	14109-72-9	1-Methylthio-2-propanone	0.075±0.017
544-35-4	Ethyl linoleate	0.104±0.027	13925-03-6	2-Ethyl-6-methyl-pyrazine	0.055±0.037
4356-52-9	Glucobrassicin	0.102±0.024	110-12-3	5-Methyl-2-hexanone	0.049±0.003
4411-89-6	2-Phenyl-2-butenal	0.088±0.021	289-95-2	Pyrimidine	0.039±0.004
56599-95-2	2-Bromooctadecanal	0.080±0.058	666-99-9	Agaricinic acid	0.040±0.0053
18409-17-1	Trans-2-Octen-1-ol	0.078±0.015	101-41-7	Methyl phenylacetate	0.08±0.0818
3268-49-3	3-(Methylthio)propionaldehyde	0.076±0.052	1003-29-8	Pyrrole-2-carboxaldehyde	0.363±0.438
124-06-1	Ethyl tetradecanoate	0.076±0.019	101-97-3	Ethyl phenylacetate	0.016±0.010
101-97-3	Ethyl phenylacetate	0.071±0.036	112-39-0	Methyl palmitate	0.041±0.013
1191-99-7	2,3-Dihydrofuran	0.067±0.030	56554-64-4	1,1-Didodecoxyhexadecane	0.032±0.017
1191-41-9	Ethyl linolenate	0.063±0.011	108-39-4	m-Cresol	0.055±0.035
5469-16-9	Dihydro-4-hydroxy-2(3H)-furanone	0.046±0.012	3737-95-9	Calconcarboxylic acid	0.033±0.021
Doujiang			**Natto**		
5617-32-3	Heptaethylene glycol	17.643±8.297	123-32-0	2,5-Dimethyl pyrazine	25.612±9.761
5117-19-1	Octaethylene glycol	6.412±2.849	14667-55-1	2,3,5-Trimethylpyrazine	10.653±1.466
3055-98-9	Octaethylene glycol lauryl ether	5.782±3.791	1124-11-4	Tetramethylpyrazine	6.780±4.741
110-44-1	Sorbic acid	5.318±3.080	5617-32-3	Heptaethylene glycol	4.866±1.397
56-81-5	Glycerol	2.768±0.947	1066-42-8	Dimethylsilanediol	2.731±0.685
541-05-9	Hexamethylcyclotrisiloxane	2.460±1.296	541-05-9	Hexamethylcyclotrisiloxane	2.513±0.561
71-41-0	1-Pentanol	2.416±2.910	20662-84-4	2,4,5-Trimethyloxazole	2.067±1.668
1066-42-8	Dimethylsilanediol	2.219±0.305	5117-19-1	Octaethylene glycol	1.745±0.394
2615-15-8	Hexaethyleneglycol	1.852±0.816	3055-98-9	Octaethylene glycol lauryl ether	1.688±0.121
64-17-5	Ethanol	1.560±1.416	513-85-9	2,3-Butanediol	1.463±0.637
513-85-9	2,3-Butanediol	1.501±1.286	64-19-7	Acetic acid	1.178±0.076
98-00-0	Furfuryl alcohol	1.389±0.678	100-52-7	Benzaldehyde	1.066±1.141
28564-83-2	Spermine tetrahydrochloride	1.329±0.273	13360-65-1	2-Ethyl-3,5-dimethylpyrazine	0.841±0.164
122-78-1	Phenylacetaldehyde	1.272±0.259	79-31-2	Isobutyric acid	0.713±0.148
100-52-7	Benzaldehyde	0.947±0.119	104-76-7	2-Ethylhexanol	0.669±0.235
628-97-7	Ethyl palmitate	0.913±0.280	110-12-3	5-Methyl-2-hexanone	0.544±0.147
3268-49-3	3-(Methylthio)propionaldehyde	0.912±0.314	565-61-7	3-Methyl-2-pentanone	0.540±0.273
79-20-9	Methyl acetate	0.784±0.228	109-78-4	3-Hydroxypropionitrile	0.519±0.374
106-32-1	Ethyl caprylate	0.540±0.167	2615-15-8	Hexaethyleneglycol	0.500±0.090
111-62-6	Ethyl oleate	0.524±0.115	100-42-5	Styrene	0.451±0.445
3391-86-4	1-Octen-3-ol	0.522±0.329	17455-13-9	18-Crown-6	0.401±0.264
140-67-0	Estragole	0.517±0.159	110-43-0	2-Heptanone	0.352±0.066
108-05-4	Vinyl acetate	0.436±0.061	5704-20-1	2-Hydroxy-3-pentanone	0.305±0.081
93-89-0	Ethyl benzoate	0.358±0.169	98-00-0	Furfuryl alcohol	0.305±0.048
5469-16-9	Dihydro-4-hydroxy-2(3H)-furanone	0.338±0.147	115-22-0	3-Hydroxy-3-methyl-2-butanone	0.264±0.108
108-95-2	Phenol	0.278±0.179	763-32-6	2-Methyl-1-buten-4-ol	0.264±0.113

96-48-0	Gamma-butyrolactone	0.266±0.072	294-93-9	12-Crown-4	0.224±0.159
71-36-3	1-Butanol	0.264±0.215	108-95-2	Phenol	0.207±0.095
108-88-3	Toluene	0.263±0.248	5076-20-0	Tetramethyloxirane	0.191±0.068
17455-13-9	18-Crown-6	0.261±0.070	18217-12-4	5-Methyl-2-heptanone	0.186±0.044
625-28-5	3-Methylbutanenitrile	0.247±0.281	629-66-3	2-Nonadecanone	0.170±0.083
79-31-2	Isobutyric acid	0.225±0.092	556-67-2	Octamethylcyclotetrasiloxane	0.166±0.063
616-45-5	2-Pyrrolidinone	0.207±0.091	108-88-3	Toluene	0.141±0.040
865-40-7	Nitrosomethane	0.186±0.177	71-41-0	1-Pentanol	0.138±0.057
3857-25-8	5-Methyl-2-furanmethanol	0.169±0.065	3555-47-3	Silicic acid tetrakis(trimethylsilyl) ester	0.131±0.156
447412-24-0	2-Amino-4,4,6,6-tetramethylthieno[2,3-c] furan-3-carbonitrile	0.136±0.087	13360-64-0	2-Ethyl-5-methylpyrazine	0.125±0.077
4356-52-9	Glucobrassicin	0.124±0.071	71-36-3	1-Butanol	0.117±0.068
3658-77-3	4-Hydroxy-2,5-dimethyl-3(2H) furanone	0.118±0.031	17151-09-6	1,2-Phenylenebis (Trimethylsilane)	0.114±0.102
870-93-9	DL-Homocystine	0.113±0.099	100-51-6	Benzyl alcohol	0.108±0.062
55282-12-7	3-Ethyl-5-(2-ethylbutyl) octadecane	0.109±0.025	66-25-1	Hexanal	0.102±0.031
600-14-6	2,3-Pentanedione	0.108±0.039	600-14-6	2,3-Pentanedione	0.083±0.036
1191-41-9	Ethyl linolenate	0.104±0.048	108-50-9	2,6-Dimethylpyrazine	0.082±0.051
21061-10-9	cis-8,11,14-Eicosatrienoic acid methyl ester	0.102±0.024	55282-12-7	3-Ethyl-5-(2-ethylbutyl) octadecane	0.080±0.042
101-97-3	Ethyl phenylacetate	0.102±0.066	13325-10-5	4-Amino-1-butanol	0.064±0.016
624-92-0	Dimethyl disulfide	0.094±0.030	4356-52-9	Glucobrassicin	0.063±0.040
3055-97-8	Dodecylheptaglycol	0.090±0.033	5469-16-9	Dihydro-4-hydroxy-2(3H)-furanone	0.046±0.010
5390-28-3	2-Nitroethanol propionate	0.061±0.021	56554-64-4	1,1-Didodecoxyhexadecane	0.025±0.011
3188-00-9	Dihydro-2-methyl-3(2H)-furanone	0.056±0.035	21061-10-9	cis-8,11,14-Eicosatrienoic acid methyl ester	0.025±0.002
5053-43-0	2-Methylpyrimidine	0.079±0.053	625-51-4	n-(Hydroxymethyl)acetamide	0.017±0.009
636-41-9	Methylpyrrole	0.030±0.012	628-97-7	Ethyl palmitate	0.012±0.003
**BBP**			**Soy sauce**		
105-54-4	Ethyl butanoate	6.697±3.920	64-17-5	Ethanol	31.027±6.68
5617-32-3	Heptaethylene glycol	5.721±0.295	5617-32-3	Heptaethylene glycol	4.434±0.333
110-44-1	Sorbic acid	5.126±1.906	123-51-3	3-Methyl-1-butanol	3.936±0.822
5117-19-1	Octaethylene glycol	3.390±0.952	2785-89-9	4-Ethyl-2-methoxyphenol	3.146±2.357
513-85-9	2,3-Butanediol	3.118±1.367	96-76-4	2,4-Di-t-butylphenol	2.614±1.160
3055-98-9	Octaethylene glycol lauryl ether	2.961±0.608	513-85-9	2,3-Butanediol	2.549±0.791
108-64-5	Ethyl isovalerate	2.780±0.626	60-34-4	Methylhydrazine	2.470±3.366
64-19-7	Acetic acid	2.663±2.711	122-78-1	Phenylacetaldehyde	2.243±0.467
541-05-9	Hexamethylcyclotrisiloxane	2.563±0.357	64-19-7	Acetic acid	2.089±2.318
7452-79-1	Ethyl 2-methylbutanoate	2.518±0.636	98-00-0	Furfuryl alcohol	2.076±0.357
123-92-2	Isoamyl acetate	2.404±1.665	556-67-2	Octamethylcyclotetrasiloxane	1.959±0.640
66-25-1	Hexanal	1.786±0.990	5117-19-1	Octaethylene glycol	1.683±0.276

123-66-0	Ethyl caproate	1.592±0.801	3055-98-9	Octaethylene glycol lauryl ether	1.563±0.408
100-52-7	Benzaldehyde	1.207±0.149	108-50-9	2,6-Dimethylpyrazine	1.393±0.803
2615-15-8	Hexaethylene glycol	1.019±0.266	31602-63-8	5-Aminomethyltetrazole	1.275±0.503
110-19-0	Isobutyl acetate	0.962±0.554	1066-42-8	Dimethylsilanediol	1.177±0.078
1066-42-8	Dimethylsilanediol	0.903±0.055	105-43-1	3-Methylvaleric Acid	1.154±0.444
98-00-0	Furfuryl alcohol	0.719±0.159	865-40-7	Nitrosomethane	1.069±1.393
626-89-1	4-Methyl-1-pentanol	0.705±0.177	541-05-9	Hexamethylcyclotrisiloxane	0.933±0.251
78-70-6	Linalool	0.646±0.054	78-83-1	Isobutanol	0.790±0.302
107-92-6	Butyric acid	0.613±0.431	71-36-3	1-Butanol	0.782±0.446
25415-67-2	4-Methyl-pentanoic acid ethyl ester	0.597±0.503	100-52-7	Benzaldehyde	0.667±0.111
122-78-1	Phenylacetaldehyde	0.562±0.211	14667-55-1	2,3,5-Trimethylpyrazine	0.631±0.190
110-43-0	2-Heptanone	0.554±0.107	13360-64-0	2-Ethyl-5-methylpyrazine	0.558±0.288
625-28-5	3-Methylbutanenitrile	0.501±0.416	123-07-9	4-Ethylphenol	0.481±0.359
503-74-2	Isovaleric acid	0.501±0.473	3391-86-4	1-Octen-3-ol	0.444±0.091
78-83-1	Isobutanol	0.464±0.187	17455-13-9	18-Crown-6	0.432±0.241
2785-89-9	4-Ethyl-2-methoxyphenol	0.431±0.174	2615-15-8	Hexaethyleneglycol	0.407±0.248
79-31-2	Isobutyric acid	0.348±0.130	123-25-1	Diethyl succinate	0.398±0.148
4356-52-9	Glucobrassicin	0.297±0.105	79-31-2	Isobutyric acid	0.389±0.276
100-42-5	Styrene	0.250±0.269	505-10-2	3-Methylthiopropanol	0.334±0.114
78-92-2	2-Butanol	0.221±0.099	13360-65-1	2-Ethyl-3,5-dimethylpyrazine	0.325±0.177
142-62-1	Hexanoic acid	0.202±0.057	3268-49-3	3-(Methylthio)propionaldehyde	0.296±0.055
3268-49-3	3-(Methylthio)propionaldehyde	0.201±0.087	625-48-9	2-Nitroethanol	0.263±0.109
3391-86-4	1-Octen-3-ol	0.192±0.039	628-97-7	Ethyl palmitate	0.209±0.006
543-49-7	2-Heptanol	0.190±0.056	106-32-1	Ethyl caprylate	0.192±0.103
1073-11-6	Lilac lactone	0.187±0.079	646-07-1	4-Methylpentanoic acid	0.189±0.130
55282-12-7	3-Ethyl-5-(2-ethylbutyl) octadecane	0.184±0.057	1192-62-7	2-Acetylfuran	0.178±0.012
79-20-9	Methyl acetate	0.178±0.058	3194-15-8	2-Propionylfuran	0.176±0.045
71-36-3	1-Butanol	0.174±0.072	101-97-3	Ethyl phenylacetate	0.165±0.049
71-41-0	1-Pentanol	0.171±0.048	104-46-1	cis-Anethol	0.164±0.092
110-93-0	6-Methyl-5-hepten-2-one	0.164±0.014	13925-06-9	2-Isobutyl-3-methylpyrazine	0.156±0.101
34314-83-5	3-methyl-4,5-dihydrofuran	0.144±0.110	112-05-0	Nonanoic acid	0.151±0.016
108-95-2	Phenol	0.126±0.026	83410-52-0	1,4,7,10,13,16,19-Heptaoxacycloheneicosan-2-one	0.144±0.092
505-10-2	3-Methylthiopropanol	0.123±0.052	1124-11-4	Tetramethylpyrazine	0.14±0.0358
100-51-6	Benzyl alcohol	0.119±0.052	5053-43-0	2-Methylpyrimidine	0.135±0.088
10348-47-7	2-Hydroxy-4-methyl-pentanoic acid ethyl ester	0.117±0.031	10348-47-7	2-Hydroxy-4-methyl-pentanoic acid ethyl ester	0.135±0.058
119-36-8	Methyl salicylate	0.109±0.031	28564-83-2	Spermine tetrahydrochloride	0.124±0.043
646-07-1	4-Methylpentanoic acid	0.103±0.014	119-67-5	2-Carboxybenzaldehyde	0.118±0.028
98-55-5	alpha-Terpineol	0.098±0.016	100-51-6	Benzyl alcohol	0.116±0.034
865-40-7	Nitrosomethane	0.096±0.061	103-79-7	Phenylacetone	0.107±0.037
21061-10-9	cis-8,11,14-Eicosatrienoic acid methyl ester	0.083±0.046	55282-12-7	3-Ethyl-5-(2-ethylbutyl) octadecane	0.106±0.010

96-48-0	Gamma-butyrolactone	0.079±0.018	3878-55-5	mono-Methyl succinate	0.104±0.036
			104-76-7	2-Ethylhexanol	0.104±0.069
			4356-52-9	Glucobrassicin	0.102±0.029
			110-12-3	5-Methyl-2-hexanone	0.098±0.042
			7786-61-0	4-Hydroxy-3-methoxystyrene	0.089±0.005
			4411-89-6	2-Phenyl-2-Butenal	0.085±0.041
			124-07-2	Octanoic acid	0.082±0.021
			6456-92-4	1,3-Dimethyl-2(1H)-Pyridinone	0.077±0.033
			111-62-6	Ethyl oleate	0.069±0.016
			2444-28-2	2,6-di-tert-butylhydroquinone	0.066±0.031
			3055-97-8	Dodecylheptaglycol	0.066±0.015
			108-88-3	Toluene	0.059±0.025
			105-42-0	4-Methyl-2-hexanone	0.059±0.031
			544-35-4	Ethyl linoleate	0.055±0.012
			21834-92-4	2-Phenyl-5-methyl-2-hexenal	0.054±0.021
			78-59-1	Isophorone	0.054±0.005

* The relative content of volatile compounds were represented as mean ± SD obtained across triplicate measurements. The table showed only the flavor compounds that were presented in three different brands of the same fermented soy food at the same time.
